# Optimizing β-Phase Content in PVDF Membranes via Modification of Dope Solution with Citric Acid/Nano-TiO_2_ Using Nonsolvent-Induced Phase Separation Method

**DOI:** 10.3390/polym17040481

**Published:** 2025-02-12

**Authors:** Md. Nahid Parvez Roni, Tanvir Ahmed Neshath, Md. Azizul Hakim, Md. Mahadi Hasan, M. Habibur Rahman, Md. Shamim Hossan, A. A. S. Mostofa Zahid, Md. Nur E Alam, Most. Halima Khatun

**Affiliations:** 1 Department of Chemistry, University of Rajshahi, Rajshahi 6205, Bangladesh; nahidparvezchem@gmail.com (M.N.P.R.); tanvirahamed01723@gmail.com (T.A.N.); azizulhbru@gmail.com (M.A.H.); s1910623126@ru.ac.bd (M.M.H.); shamim.chem@ru.ac.bd (M.S.H.); zahid@ru.ac.bd (A.A.S.M.Z.); 2 Bangladesh Atomic Energy Commission, Dhaka 1207, Bangladesh; mdnur_e_alam@yahoo.com; 3 BCSIR Rajshahi Laboratories, Rajshahi 6026, Bangladesh; halimakhatun@bcsir.gov.bd

**Keywords:** poly(vinylidene fluoride), NIPS, nano-TiO_2_, citric acid, electroactive polymer, β-polymorph

## Abstract

The morphology of Poly (vinylidene fluoride) (PVDF) membranes prepared via the nonsolvent-induced phase separation (NIPS) method was modulated by altering the dope solution with citric acid (CA) and titanium dioxide nanoparticles (nano-TiO_2_) to optimize the β-phase content. Three series of dope solutions were prepared in dimethyl acetamide (DMAc): (i) TONx series contained 0.0–10% citric acid, (ii) Mx series contained 0.0–0.4% nano-TiO_2_, and (iii) TAx series contained 5% CA and 0.0–0.40% nano-TiO_2_. A field emission scanning electron microscopy (FESEM) study revealed that CA enhances pore opening, and nano-TiO_2_ transforms the sponge-like uneven porous structures into a compact, relatively regular honeycomb structure in the PVDF membranes. The combined effect of CA and nano-TiO_2_ in the dope solution made the channels and chambers of the membrane well organized, and the walls of the channels transformed from solid fibrils to cross-woven nanofiber-like entities. Porosity initially peaked at 84% in the TAx series, gradually decreasing to 72% with increasing nano-TiO_2_ concentrations. X-ray diffraction (XRD), Fourier-Transformed Infrared Spectroscopy (FTIR), and Differential Scanning Calorimetry (DSC) revealed the presence of a combined relative amount of the β- and γ-polymorphs of 84% in a neat PVDF membrane, 88% in an Mx, and 96% in a TAx series membrane, with the β-PVDF constituting nearly the entire portion of the combined polymorphs. The presence of 96% electroactive polymorph content in the PVDF membrane is noteworthy, highlighting its potential biomedical and industrial applications.

## 1. Introduction

Contemporary research in membrane technology focuses on enhancing membrane characteristics for various purposes, including separation, purification, drug encapsulation, energy production, and sensing in diverse disciplines, including industrial applications, environmental practices, and human health [1,2,3,4,5]. These efforts intend to improve the efficiency and productivity of membranes and promote sustainability and cost-effective solutions [6]. The advancement of novel materials like electroactive polymers is essential within this framework. Their flexible characteristics and easy adaptability for specific applications provide unique opportunities in the sustainable energy sector [7]. Polyaniline (PANI), Polypyrrole (PPy), Poly(vinylidene fluoride) (PVDF), Polypropylene (PP), Polythiophene (PTh), Polyacetylene (PAc), and Nafion are some of the widely used electroactive polymers [8,9]. Their exceptional electrical characteristics and adaptability have drawn significant attention in various applications, including energy storage and production, actuators, sensors, and spintronics [10,11]. Again, an electroactive polymer with biocompatibility would make the material an ideal candidate for application in biomedical devices, prostheses, acoustic transducers, actuators, medical imaging, wearable devices, or smart textile and intelligent scaffolds for tissue engineering [12,13,14]. Among the various electroactive polymers, PVDF is distinguished by its exceptional piezoelectric, pyroelectric, and ferroelectric properties [15], making it particularly suitable for many of the abovementioned applications.

PVDF, with the repeating unit –CH_2_–CF_2_–, is a highly versatile polymer membrane material due to its unique combination of desirable properties. These include robust mechanical strength [16], excellent resistance to oxidation, high thermal and hydrolytic stability, chemical and thermal inertness, flexibility [12], and superior electrical characteristics (like piezoelectricity and pyroelectricity) [10]. PVDF is semicrystalline [17], and what makes it even more significant is its range of crystalline polymorphs, α, β, γ, δ, and ε, each with distinct molecular arrangements and associated properties [18]. The most thermodynamically stable form is the α-phase PVDF, which has a nonpolar molecular chain structure with an alternating trans-gauche (TGTG′) conformation sequence of the carbon atoms along the chain. This configuration makes it unsuitable for electroactive applications.

In contrast, the β-phase of PVDF, characterized by its all-trans (TTTT) molecular arrangement, exhibits the highest polarity and electroactive polar dipole moment. The latter property gives PVDF remarkable piezoelectric, pyroelectric, and ferroelectric characteristics. The γ-phase, formed by a partially polar TTTGTTTG′ conformation, shows a moderate level of electroactivity and frequently coexists with the β-phase under specific processing conditions. The δ-phase, a polar variant of the α-phase produced under intense electric fields, has limited practical applications due to its intrinsic instability. Finally, the ε-phase occurs less often and has a conformation similar to the γ-phase but exhibits nonpolar characteristics [5]. Among these polymorphs, the β-phase of PVDF is the most desirable for electroactive applications. However, obtaining higher β-phase content in PVDF membranes poses a considerable challenge, as the polymer inherently tends to crystallize in the nonpolar α-phase during conventional processing. Researchers have investigated multiple strategies to address this limitation, such as modifying fabrication parameters (e.g., concentration, temperature, evaporation rate, nature of the precipitation bath, and solvent type) [19], incorporating additives, and applying advanced processing methods [20].

PVDF membranes are commonly fabricated via phase separation methods induced by temperature (TIPS) [21,22], nonsolvent (NIPS) [19], vapor (VIPS) [23], and evaporation (EIPS) [24] or a combination thereof [25,26]. Moreover, nanofiber membranes can be fabricated via electrospinning [27], solution blow spinning (SBS) [15], spin-coating, and the Langmuir–Blodgett (LB) technique [28]. Several post-processing treatments such as hot pressing, quenching, annealing, solvent evaporation, stretching, rolling, plasma treatment, UV treatment, and combinations of these processes were employed to enhance the proportion of the β-phase within PVDF [29,30]. These techniques yield membranes with varying polymorphs of PVDF [31]. Among the phase separation techniques, NIPS stands out for its versatility, simplicity, cost-effectiveness, and scalability, making it highly suitable for a wide range of industrial applications [32]. Furthermore, the NIPS method allows for controlled modification of membrane structure and pore size by adjusting the composition of the precipitation bath [19].

Along with the fabrication method, choosing an appropriate solvent is crucial to optimize the production process of PVDF membranes with desired properties and performance for specific applications, as the solvent quality dictates the solubility of the polymer, its interaction with the coagulation bath, diffusion rates of solvent and nonsolvent into the dope solution, and the crystallization process, all of which influence the final morphology of the membrane, its porosity, pore size distribution, and crystallization of PVDF [25,33,34]. *N*,*N*-dimethylacetamide (DMA), *N*,*N*-dimethylformamide (DMF), 1-methyl-2-pyrrolidone (NMP), methyl ethyl ketone (MEK), tetrahydrofuran (THF), and dimethyl sulfoxide (DMSO) are some of the common solvents for PVDF membrane preparation [35,36]. It was reported that the preparation of the PVDF membrane via phase separation method with high dipole moment solvents like hexamethylphosphoramide (HMPA) favors all-trans (TTTT) conformations, yielding β-phase crystals [25]. In contrast, lower dipole moment solvents such as triethylphosphate (TEP) promote TGTG′ conformations, resulting in α-phase crystals. Solvents with intermediate polarity, e.g., DMAc and NMP, induce trans-trans-trans-gauche (TTTG) conformations, forming γ-phase crystals [25]. Moreover, temperature plays a vital role in modifying the morphology and structure of the PVDF membranes. Thus, α-crystalline structure predominates in PVDF/TiO_2_ membrane when fabrication occurs at 50 °C and 70 °C. Conversely, the β crystalline structure becomes more prevalent at lower fabrication temperatures of 10 °C and 25 °C [37]. The PVDF and PVDF/TiO_2_ membranes exhibit reduced porosity with rising temperatures. Thus, the porosity of the PVDF/TiO_2_ membranes declines from 83% at 10 °C to 70% at 70 °C. Similarly, the porosity of the pure PVDF membranes decreases from 78% at 10 °C to 70% at 70 °C [37].

Finally, modifying PVDF concentration and incorporating additives (polymer, organic, or inorganic nanoparticles) in the dope solution may change the morphology of the PVDF membranes dramatically [27]. Several studies have qualitatively reported the appearance of the electroactive β-phase by incorporating additives such as carbon nanotubes (CNTs) [38], magnetic nanoparticles (CoFe_2_O_4_, NiFe_2_O_4_, Fe_3_O_4_) [39,40,41], silver nanowires (AgNWs) [42], graphene [43], tetramethylammoniumchloride (TMAC) [44], zeolites [45], lithium chloride (LiCl) [46], titanium dioxide (TiO_2_) [47], zinc oxide (ZnO) [48], barium titanate (BaTiO_3_) [48], potassium sodium niobite (KNN) [49], nitrate salt [50], and piezoelectric ceramic particles. A handful of articles have reported quantitative estimates of the β-phase crystallinity of PVDF in the membrane. Most of these works induced the electroactive phase by post-treatment of the membranes; a summary of their findings is presented in Table 1.

Among all of the additives, titanium dioxide (TiO_2_) is mentionable due to its antifouling, antibacterial, and photocatalytic properties [47,57,58] and potentiality as a biocidal material [59,60,61]. González-Benito et al. reported the presence of the β-phase in membranes with 10% (*w*/*v*) nano-TiO_2_ incorporation as the nanoscopic nature of the particles permits their incorporation in minimal quantities and their substantial surface-to-volume ratio results in a large interphase that alters the characteristics of the polymer matrix [62]. On the other hand, Zhang et al. mentioned the preparation of the PVDF/tributyl citrate (TBC) membrane. They observed that increasing the amount of PVDF from 36 wt.% to 40 wt.% results in less porous membranes with smaller average pore sizes and a more pronounced crystalline structure [63]. In addition, Adebayo et al. showed that the citric acid-coated Fe_3_O_4_/PVDF membrane (40 wt.%) exhibit effective microwave absorption properties across the explored frequency range (8.2–18 GHz) [64].

The literature review reveals that prior research has predominantly examined the isolated impact of nano-TiO_2_ on PVDF membranes. However, there is a lack of systematic investigation into the individual role of citric acid and the combined effects of nano-TiO_2_ and citric acid in the dope solution for the NIPS technique. The literature also lacks quantitative data on the electroactive polymorph content in PVDF membranes. This work aimed at preparing high β-phase-containing PVDF membranes for potential biomedical and industrial applications by systematically investigating the combined effects of citric acid and nano-TiO_2_ in the dope solution on the structural and morphological characteristics of PVDF membranes. We found that the combined effect of citric acid and nano-TiO_2_ in the dope solution resulted in the crystallization of PVDF into electroactive polymorphs to 96% in a membrane. This result demands a detailed investigation of individual and combined impacts of these additives on the morphology, porosity, and β-phase crystallinity of PVDF membranes.

## 2. Experimental Section

### 2.1. Materials and Reagents

Titanium tetraisopropoxide (TTIP) was a product of TCI, Japan; Poly(vinylidene fluoride) was purchased from Alfa Aesar, USA (CAS number: 24937-79-9) with a melt viscosity of 26.22 kilo poise, the molecular weight of which was ${\bar{M}}_{v}≅{\bar{M}}_{w}=$ (2.64 ± 0.29) × 10^5^ g/mol, determined by intrinsic viscosity measurements in dilute dimethyl acetamide (DMAc, from SRL India) solutions at 25 °C. The water used had a TDS < 3 ppm obtained by a locally assembled multistage combined reverse osmosis membrane–ion exchange resin filtration system. All other chemicals used were of analytical grade and used without further purification.

### 2.2. Preparation of PVDF Dope Solutions

The TiO_2_ nanoparticles (synthesized via a sol–gel method using TTIP, as described elsewhere [65]) of a desired weight were stirred continuously for 24 h at ambient temperature (25 °C) with DMAc of a definite volume (typically 10–15 mL) to obtain a stable dispersion (step 1). Then, a desired amount of PVDF was added to the mixture, with the stirring continued for a further 24 hours at room temperature to obtain a stable 10% (*w*/*v*) PVDF/TiO_2_ dope solution (step 2). This dope solution was then placed in an ultrasonic bath at 60 °C operated in the degassing mode to eliminate air bubbles (step 3). Eight different dope solutions (DS-1) of the polymer containing 0.0–0.4% (*w*/*v*) nano-TiO_2_ were prepared. A second series of the PVDF dope solutions was prepared by adding a weighed amount of citric acid (CA) at the end of step 2 to make the dope solution 5% in citric acid (DS-2). A third series of PVDF dope solutions was prepared in the same manner without any nanoparticles but with varying amounts of CA to make them 0–10% (*w*/*v*) in citric acid (DS-3). Table 2 lists the components of the PVDF dope solutions and the designation of the membranes prepared from them.

### 2.3. Fabrication of PVDF Membrane Films

The dope solution was cast onto clean, dry, and spotless glass slides by sliding a stainless-steel film applicator (300 µm gap) over it. Then, it was placed in a 1:2.3 (*v*/*v*) solution of isopropanol and deionized (DI) water for 10 min, after which the membrane-containing slide was placed in a DI water bath for 24 h. Finally, the membrane was dried under vacuum in a vacuum desiccator containing fused CaCl_2_ at ambient temperature (25 °C). The thickness of the membranes ranged between 30 and 45 µm, measured by a micrometer screw gauge.

### 2.4. Characterization

The surface and cross-sectional structure of PVDF nanocomposite membranes were studied by using a field emission scanning electron microscope (FTSEM, JEOL JSM-IT800, Tokyo, Japan) at an accelerating voltage of 15 kV.

The porosity of the membranes was measured by the gravimetric method [66] using hexanol-1 as the penetrating medium. Suitable pieces of PVDF membranes were immersed in hexanol-1 for several minutes, after which the excess alcohol on the surfaces of the films was sucked up using filter paper. The membranes were weighed in wet and dry conditions by a semi-micro analytical balance, and the porosity was calculated using Equation (1).

(1) $$e=\frac{\frac{w_{2}-w_{1}}{\rho_{a}}}{\left(\frac{w_{2}-w_{1}}{\rho_{a}}+\frac{w_{1}}{\rho_{p}}\right)}\times 100$$

where w_1_ is the mass of the dry membrane, w_2_ is the mass of the wet membrane, $\rho_{p}$ is the density of PVDF, and $\rho_{a}$ is the density of hexanol-1.

A PerkinElmer model FTIR-100 infrared spectrometer was used to analyze the PVDF powder and the composite membranes for the identification and quantitative estimation of different polymorphs of PVDF in the membranes. The FTIR spectra were scanned over the wave number range 4000 cm^−1^ to 350 cm^−1^ with 2.0 cm^−1^ intervals. The percentage of β- and γ-polymorphs of the PVDF in powder and the nanocomposite membranes was calculated via the Gregorio method [67]. The Gregorio method is based on the comparison of the absorption peak of the α-phase at 763 cm^−1^ [68] and of β- + γ-phases at 840 cm^−1^ [69], which can be derived from Beer–Lambert law.

(2) $$F_{\beta +\gamma }=\frac{X_{840}}{X_{763}+X_{840}}=\frac{A_{840}}{\left(\frac{K_{840}}{K_{763}}\right)A_{763}+A_{840}}=\frac{A_{840}}{1.3A_{763}+A_{840}}$$

where the value of the absorption coefficient K_763_ is 6.1 × 10^4^ cm^2^/mole and that of K_840_ is 7.7 × 10^4^ cm^2^/mole; and A_763_ and A_840_ are absorption intensities corresponding to the absorption peaks at 763 cm^−1^ and 840 cm^−1^, respectively. The absorption intensity of the corresponding peaks was determined after the deconvolution of the individual absorption peaks with the aid of the OriginLab Origin 2019b data analysis and graphing software.

The X-ray diffraction analyses were performed on a SmartLab-SE XRD instrument from Rigaku, Tokyo, Japan. The degree of crystallinity of the synthesized membranes was measured from the XRD data [70,71] by using Equation (3).

(3) $$\lambda \left(\%\right)=\frac{A_{c}}{A_{c}+A_{a}}\times 100\%$$

where A_c_ is the combined area covered under the crystallization peaks, and (A_c_ + A_a_) is the total area covered under all the peaks.

The thermal stability of the membranes was assessed using PerkinElmer STA-6000, a simultaneous TG/DTA instrument. The thermograms were recorded in the temperature range of 30 °C to 800 °C at a scan rate of 10 °C/min under the nitrogen gas flow.

A Perkin–Elmer DSC-8000 instrument fitted with an Intracooler-2 system was utilized to investigate the crystallization and melting characteristics of the composites. Successive heating and cooling runs were performed from 50 °C to 190 °C interspersed by a one-minute isothermal step after each heating or cooling run. All heating scans were carried out at the rate of 20 °C/min, while the cooling scans after the short isothermal step were carried out at the rate of 10 °C/min. The degree of crystallinity of the PVDF membrane was calculated from DSC data [71,72] by applying Equation (4).

(4) $$X_{C}=\frac{{∆H}_{f}/\varphi }{{∆H}_{f}^{*}}$$

where ΔH_f_ is the enthalpy of fusion of the DSC samples; ΔH_f_* is the standard enthalpy of fusion of PVDF, which depends on the relative percentage of various polymorphs of PVDF; and φ is the weight fraction of PVDF in PVDF-nanocomposites.

The fusion enthalpy of 100% pure α-polymorph of PVDF was obtained from the literature to be ${∆H}_{\alpha }^{0}=104.5\;J/g$, and that for 100% pure β polymorph is ${∆H}_{\beta }^{0}=219.7\;J/g$, which was used for the calculation of the combined β- and γ-polymorphs (i.e., as ${∆H}_{\beta +\gamma }^{0}$). With this value, the standard enthalpy of fusion [73] was calculated utilizing Equation (5).

(5) $${∆H}_{f}^{*}={∆H}_{\alpha }^{0}.X_{\alpha }+{∆H}_{\beta +\gamma }^{0}.X_{\beta +\gamma }$$

where X_α_ is the fraction of α polymorph, and X_β+γ_ is the fraction of (β + γ)-polymorph in a sample. X_α_ and X_β+γ_ were measured from FTIR data.

## 3. Results and Discussion

### 3.1. Characterization of TiO_2_ Nanoparticles

**Figure 1:**
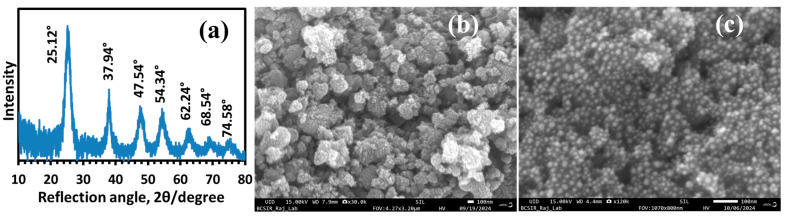
(**a**) XRD Pattern of nano-TiO_2_; (**b**,**c**) FESEM images of corresponding particles at 30 k and 120 k magnification, respectively, with 100 nm scale bar, taken using a JEOL model no. JSM IT800 FESEM operated at 15.00 kV. The average aggregate size is 159 ± 7 nm (sample size, n = 107) in (**b**), and the average nanoparticle size is 19.4 ± 0.3 nm (n = 231) in (**c**).

The size, morphology, and crystalline structure of synthesized TiO_2_ nanoparticles were characterized by XRD and FESEM techniques. The X-ray powder diffraction (XRD) pattern of the TiO_2_ nanoparticles in Figure 1a displays strong diffraction peaks at 2θ = 25.12°, 37.94°, 47.54°, 54.34°, 62.24°, 68.54°, and 74.58°, which represent the planes (101), (004), (200), (105), (204), (116), and (215), respectively, of the tetragonal anatase form of TiO_2_ [74,75]. The degree of crystallinity of the TiO_2_ nanoparticles was calculated from XRD data to be 36.7%. The crystallite size determined by the Williamson-–Hall plot [76] was 2.94 nm, while by the Sherrer equation [77], it was 4.60 nm. The high-resolution FESEM images of the nanoparticles used in the study display aggregates of the nanoparticles. The analysis of the SEM image by the ImageJ software gave an average aggregate size of 159 ± 7 nm and an average nanoparticle size of 19.4 ± 0.3 nm.

### 3.2. Characterization of Commercial PVDF Powder

The commercial PVDF in powder form utilized in this research was characterized using XRD, FTIR, DSC, and intrinsic viscosity measurements.

The molecular weight of the commercial PVDF powder was determined by intrinsic viscosity measurements in DMAc solution at 25 °C using the Mark–Houwink–Sakurada equation that relates the intrinsic viscosity, $\left[\eta \right]$, with the viscosity average molecular weight in the following manner:

(6) $$\left[\eta \right]=K{{\bar{M}}_{v}}^{\alpha }$$

where the values of K and α for the PVDF−DMAc system at 25 °C were 2.01 × 10^−4^ dL/g and 0.675, respectively, as found in the literature [78]. The viscosity average molecular weight, an estimate of the weight-averaged molecular weight of the PVDF powder, was found to be (2.64 ± 0.29) × 10^5^ g/mol, confirming the material to be a high polymer.

**Figure 2:**
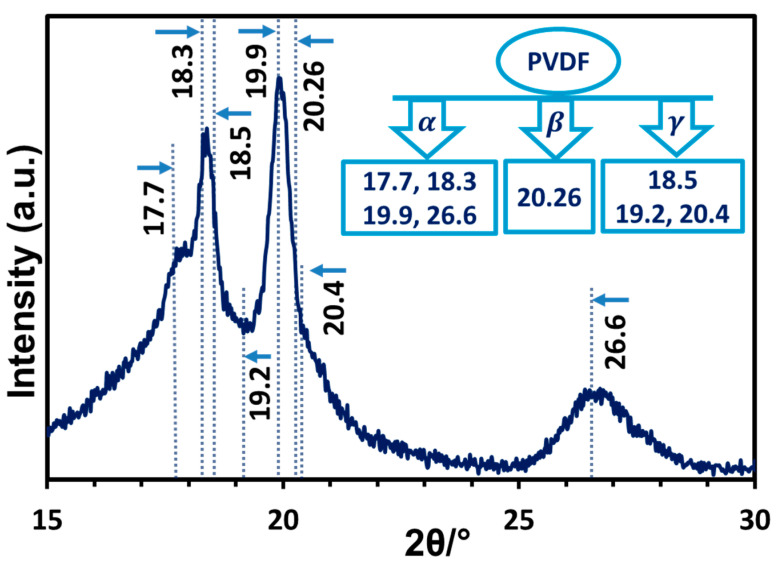
XRD pattern of commercial PVDF powder used to prepare nanocomposite membranes. Inset: Most prominent diffraction positions (2θ) characteristic of different PVDF polymorphs (indicated by arrows in graph).

In XRD, the pure α-phase shows characteristic diffraction peaks at angles (2θ) 17.7°, 18.3°, 19.9°, and 26.6°. In comparison, the β-phase shows a distinct diffraction peak at 20.26°, while the γ-phase shows peaks at 18.5°, 19.2°, and 20.4° [20]. Figure 2 displays the XRD pattern of the commercial PVDF powder where sharp diffraction peaks characteristic of the pure alpha polymorph are present. The degree of crystallinity of the PVDF powder obtained from the XRD data was 34%, and the crystallite (lamellar) size measured by the Williamson–Hall [76] plot was 48 nm.

**Figure 3:**
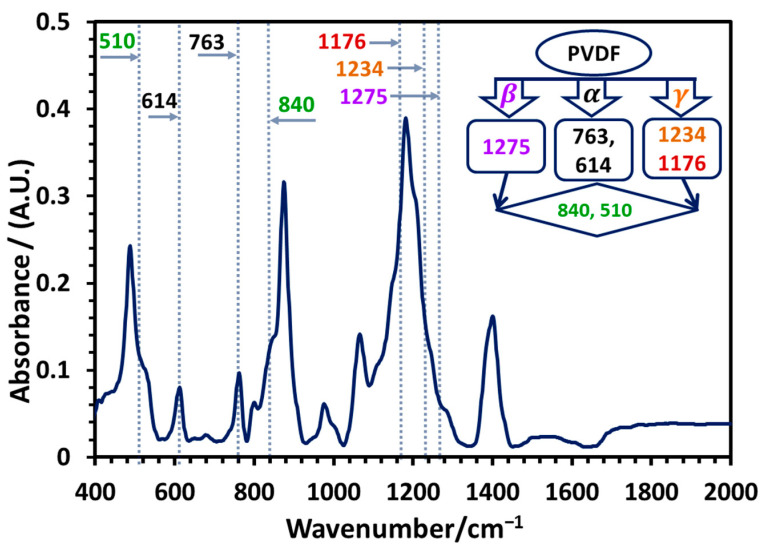
FTIR spectra of commercial PVDF powder used for preparation of nanocomposite membranes. Inset: Most prominent characteristic IR absorption frequencies (in cm^−1^) of different PVDF polymorphs [68].

FTIR analysis is the most convenient and reliable method for identifying and quantifying the three major polymorphs in PVDF. In FTIR spectra, the all-trans (TTTT) conformation of the PVDF β-polymorph displays a characteristic absorption at 1275 cm^−1^ due to CF_2_ stretching vibration and C-C skeleton vibration [79]. The absorption band around 840 cm^−1^, which is due to the absorption of the total deformation vibration of CH_2_ and the asymmetric stretching vibration of CF_2_ [79] and that around 510 cm^−1^, which is due to the bending and wagging modes of CF_2_ and C(F)-C(H)-C(F) skeletal bending, were assigned to be characteristic of the combined β and γ-polymorphs [55]. The alternating TGTG′ conformation (α-polymorph) shows characteristic absorptions around 763 cm^−1^ and 614 cm^−1^ due to CF_2_ bending and skeletal bending of C(F)-C(H)-C(F) [55]. In Figure 3, the sharp absorption peaks at 614 cm^−1^ and 763 cm^−1^ due to the α-phase and very low-intensity shoulders at absorption positions of the electroactive β- and γ-phases indicate the presence of a high content of α-polymorph PVDF in the sample, which also supports the XRD analysis discussed above [68]. The analysis of the spectra by the Gregorio method [67] that compares the peak intensity at 763 cm^−1^ for the α-phase with that at 840 cm^−1^ of the combined intensity of the electroactive phases shows that the commercial PVDF powder used in the study was about 84% α-polymorph.

**Figure 4:**
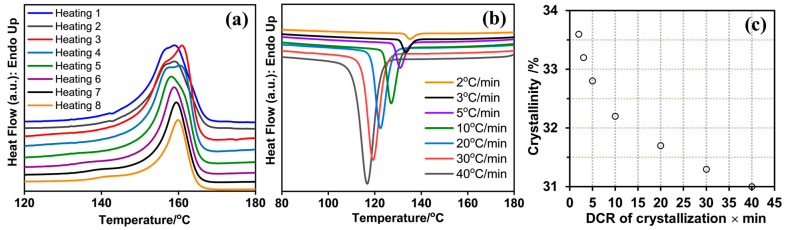
(**a**) DSC heating thermograms from 50 °C to 190 °C at 20 °C/min: dual melting peak of as-received PVDF powder (Heating 1) gradually changes to single endotherm as sample was crystallized in DSC under decreasing dynamic cooling rates (2–8). (**b**) DSC cooling thermograms of commercial PVDF powder. Sample was melted to 190 °C, equilibrated under nitrogen atmosphere, and dynamically cooled at different rates, as shown in legend. (**c**) Crystallinity (±0.1%) of PVDF formed under different dynamic cooling rates (DCRs).

The melting and crystallization characteristics of the commercial PVDF powder were studied by DSC. The DSC heating thermogram of the as-received polymer (Figure 4a, Heating 1) exhibited typical characteristics of semicrystalline PVDF, featuring a broad melting peak at 160 °C and a shoulder at the lower temperature end of the endotherm. The degree of crystallinity of the as-received polymer was 35% calculated from the enthalpy of fusion obtained from DSC; taking the enthalpy of fusion of pure α-phase and that of the (β + γ)-phase from the literature [73], the relative amounts were obtained from the FTIR data. The DSC data closely matched the degree of crystallinity of the PVDF powder obtained from the XRD data discussed earlier.

A wide range of DSC melting temperatures of PVDF was reported in the literature, ranging between 155 °C and 192 °C [20]. The melting temperature of PVDF depends on several factors, including thermal history, the polymorphic phases of PVDF crystals (α-, β-, and γ-polymorphs), and also on the regio-irregular structures along the chain [71,72]. As the polymer crystals were 84% α-polymorph, the relatively lower melting point of the polymer may suggest that it had a relatively higher degree of regio-irregularity in the structure [80,81].

After melting the as-received polymer powder in the DSC at 20 °C/min, the liquid was allowed to equilibrate at 190 °C for one minute. Subsequently, we performed a cooling scan at the rate of 40 °C/min down to 50 °C, equilibrated the sample at 50 °C in the DSC for one minute, and carried out a heating scan at 20 °C/min (Figure 4a, Heating 2). To observe the effect of the thermal history (rate of dynamic cooling) on the melting temperature of the polymer crystals formed, we repeated the crystallization process under dynamic cooling rates of 30 °C/min, 20 °C/min, 10 °C/min, 5 °C/min, 3 °C/min, and 2 °C/min. The cooling thermograms are shown in Figure 4b, and the subsequent heating thermograms are shown in Figure 4a (heating 3 to 8, respectively). Because the T_g_ of the polymer is much lower (in the range of −40 °C to −30 °C) [20], the crystallization of the polymer is supposed to be complete during the cooling run and subsequent annealing at 50 °C in the DSC. Therefore, any difference in the melting temperature might be related to the conformation of the polymer chains locked in the crystal lattice during its formation at different cooling rates and the annealing of the thicker un-melted crystals during the heating run. We noted that the melting peak gradually merged with the lower temperature shoulder of the heating thermograms as the crystals were formed at gradually lower cooling rates. Finally, a single endotherm was observed for the melting of crystals formed at the cooling rate of 5 °C/min and lower. We also noted that only a single crystallization exotherm formed for all the dynamic cooling rates studied, as shown in Figure 4b. These findings may suggest that the morphology of the crystals formed was the same. So, annealing of the un-melted crystals during the heating run might be the cause for the formation of a lower temperature shoulder (due to the melting of thinner lamella) and a higher temperature peak (due to the melting of annealed lamella) in the melting endotherms for crystals formed under dynamic cooling rates higher than 5 °C/min. The rate of dynamic cooling influenced the crystallinity formed as the crystallinity increased with decreasing cooling rates (DCRs), as observed in Figure 4c.

### 3.3. Effect of Citric Acid in Dope Solution on Morphology of PVDF (TONx Series) Membranes

The PVDF membranes were prepared using a nonsolvent-induced phase separation method from a 10% (*w*/*v*) dope solution of the polymer in DMAc. Zero to ten percent (*w*/*v*) citric acid (CA) was added to the dope solution (Table 2, DS-3) to study the effect of citric acid in the dope solution on the morphology of the PVDF membranes (TONx series). Details of the procedure is outlined in the Experimental Section. As citric acid and DMAc are highly soluble in water, subsequent washing of the membranes with deionized water removes them from the membranes, and we obtain membranes of neat PVDF. The presence of varying amounts of CA in the dope solution was expected to alter the morphology of the membranes produced.

#### 3.3.1. X-Ray Diffraction Analysis of TONx Series PVDF Membranes

**Figure 5:**
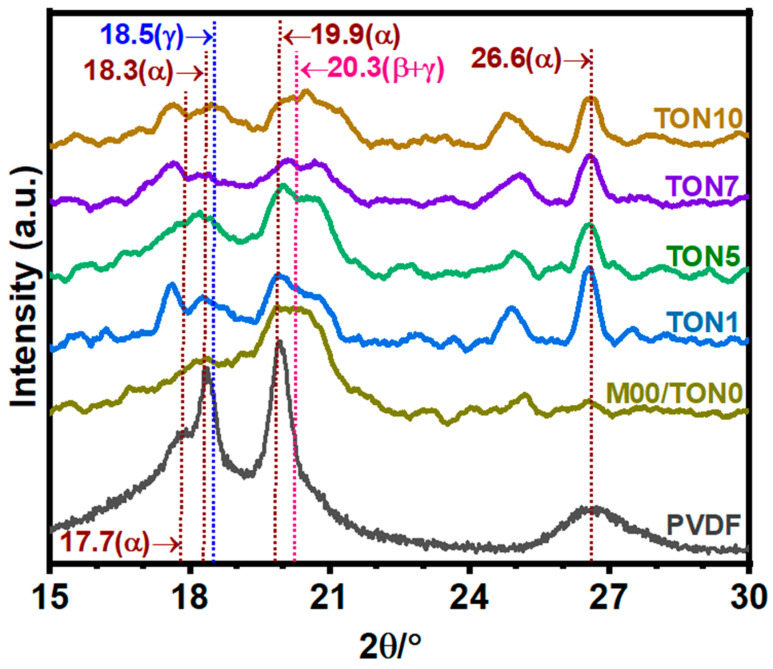
The representative XRD patterns of the PVDF membranes of the TONx series; the number (x) represents the percentage of citric acid in the PVDF dope solution used to prepare the membranes. The pattern marked PVDF represents commercial PVDF powder.

Figure 5 shows the representative XRD patterns of the PVDF membranes of the TONx series. The XRD profile of the PVDF membranes differed significantly from that of the commercial PVDF powder. The sharp absorption maxima characteristic of the α-polymorph PVDF was absent in the XRD profiles of the membranes. However, they displayed relatively broad diffraction maxima, signatures of the electroactive β- and γ-polymorph PVDF, and relatively much lower intensity diffraction maxima of the α-polymorphs, as marked in Figure 5. It is, however, interesting to note that all the membranes contain a diffraction maximum around 2θ = 20.3°, which we consider to be due to the combined reflections from (110) and (200) crystallographic planes of β-PVDF (2θ = 20.26°) and (101) planes of γ-PVDF (2θ = 20.3°) [82]. It is also interesting to note that in the TON0/M0.0 membrane, the diffraction peak of the α-polymorphs almost disappeared, but as we added CA in the dope solution, the diffraction peaks of the α-PVDF at 2θ = 26.6° reappeared. The detailed quantitative estimates of the different polymorphs were obtained from the analysis of the FTIR data of the membranes, which will be discussed in the next section. The crystallinity of PVDF in the membranes obtained from the XRD data, along with the corresponding data from DSC analysis are presented in Table 3, where it is evident that the crystallinity of PVDF declined heavily in the membranes. The crystallinity decreased because the membranes were prepared under the phase inversion process, a nonequilibrium phenomenon. It is obvious that the low level of crystallinity of PVDF in the membranes is the cause of the broadness of the XRD peaks.

#### 3.3.2. FTIR Analysis of TONx Series PVDF Membranes

**Figure 6:**
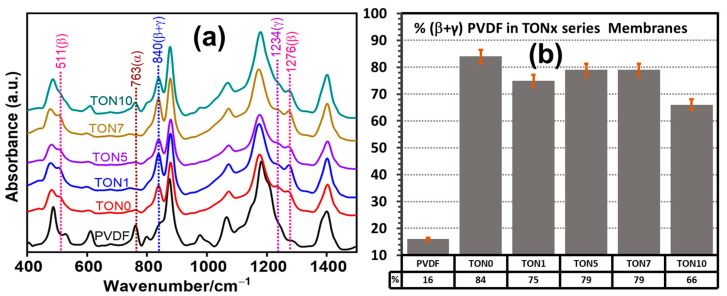
(**a**) FTIR spectra of PVDF membranes of TONx series (x is the percent of CA in dope solutions); spectra marked PVDF represent commercial PVDF powder. (**b**) Percentage (±3%) of electroactive (β + γ) polymorphs of PVDF in membranes obtained by comparing intensity of FTIR absorption bands corresponding to α-polymorph at 763 cm^−1^ and combined electroactive polymorphs (β + γ) at 840 cm^−1^, according to Gregorio Method.

**Figure 7:**
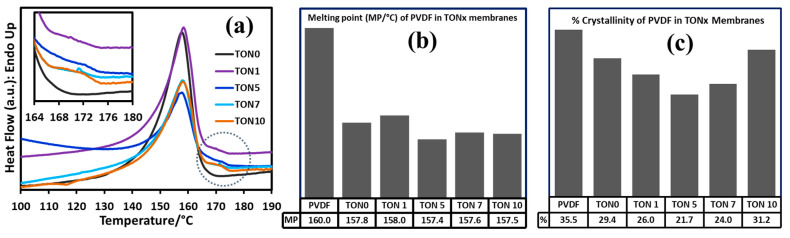
(**a**) Representative DSC heating thermograms of as-prepared TONx series PVDF membranes at 20 °C/min. Inset: Magnified view of higher temperature shoulder of melting endotherm, indicative of melting of γ-PVDF, (**b**) melting points (±0.1 °C), and (**c**) Crystallinity (±0.1%) of PVDF in membranes corresponding to endotherms in (**a**).

Figure 6a displays the FTIR spectra of the TONx series membranes. The characteristic absorption band positions of the three significant polymorphs of PVDF were marked in the spectra. We may note here that while in the commercial PVDF powder, the band at 840 cm^−1^, common to both the electroactive β and γ-phases, appeared only as a shoulder due to its very low-intensity, it displayed a distinct band in all the membranes of the TONx series (including the one prepared from the neat PVDF dope solution), clearly indicating the presence of high content of the electroactive phases in the membranes. A careful observation of Figure 6a revealed that the absorption band characterizing the individual β-phase (TTTT) at 1276 cm^−1^ was quite distinct, while that for the individual γ-phase (TTTG) PVDF at 1234 cm^−1^ appeared as a low-intensity shoulder, implying that the major part of the intensity of the band at 840 cm^−1^ was due to the strongest electroactive β-polymorph of PVDF. We can also rule out the formation of any significant amount of the γ-phase PVDF from the DSC melting thermograms (inset of Figure 7a), where we find that the melting point of PVDF in the TONx series membranes did not exceed 158 °C (Table 3), as it is well known that the melting point of the γ-phase is more than 10 °C higher than the other two abundant phases [20]. However, a careful examination of Figure 7 would reveal the presence of a shoulder at the higher temperature region of the melting endotherms of the membranes that contained CA in the dope solution (inset of Figure 7a), signifying the formation of a small proportion of the γ PVDF in these membranes. Quantitative estimates of the electroactive phases in the membranes were obtained by comparing the intensity of the band at 840 cm^−1^ to that at 763 cm^−1^ following the Gregorio method, and results are presented in Figure 6b, which reveal that the membranes of the TONx series contained a substantial proportion of the electroactive β-polymorph. However, the membrane from the neat PVDF dope solution (TON0) had the highest (84%) electroactive polymorph content, and incorporating CA in the dope solution reduced it to some extent.

It is interesting to note here that while in the PVDF powder, the electroactive polymorph was only 16%; under the condition of phase inversion, the conformation of PVDF in the membrane dramatically changed mainly to the electroactive β-polymorph to the extent of 84%, as shown in Figure 6b. Incorporating CA in the dope solution initially dropped the electroactive polymorph content slightly below 80% and remained constant at that level up to 7% CA incorporation in the dope solution. Further increase in CA in the dope solution caused the electroactive polymorph to decline to 66% for the TON10 membrane.

#### 3.3.3. Thermal Analysis of PVDF Membranes of TONx Series

TGA of the TONx series membranes (T_D_ data shown in Table 3) showed that the membranes were thermally stable up to 440 °C, proving their ability to perform at high temperatures. There is a slight decrease (maximum of 6 °C) in the degradation onset temperature of the membranes of the TONx series, presumably due to the residual trace amount of CA in the membranes.

Figure 7 displays the DSC heating thermograms of the as-prepared membranes of the TONx series, and we have presented the corresponding DSC melting and crystallinity data in Table 3. The crystallinity from the DSC data was consistent with the corresponding XRD data shown in the same table. It was observed that the crystallinity of PVDF decreased heavily in membranes compared to the commercial PVDF powder from which they were fabricated, showing a minimum crystallinity value at an intermediate concentration of citric acid in the dope solution. The melting point of PVDF in the membranes also decreased by about 2.5 °C, presumably due to the formation of thinner crystal lamella associated with the nonequilibrium phase inversion procedure of the membrane formation. The melting points of PVDF in the membranes fall in the melting range of the α- and β-phase crystals, as is consistent with the phase quantification from the FTIR spectral data (cf. Figure 6). The shoulder at the higher temperature region (around 170 °C) of the melting endotherms for all the membranes of the TONx series except M0.0/TON0 indicates that the presence of CA in the dope solution induces the formation of some γ-crystals in the membranes, which is also consistent with the XRD and FTIR spectral data shown in Figure 5 and Figure 6, respectively.

#### 3.3.4. FESEM of PVDF Membranes of TONx Series

**Figure 8:**
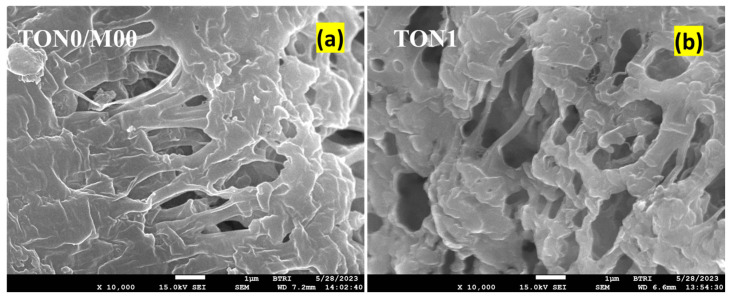
Representative FESEM images of fracture surfaces of (**a**) TON0 (neat PVDF dope solution) and (**b**) TON1 (dope solution fortified with 1% citric acid) membranes. Scale bar = 1 µm.

FESEM was used to examine the microstructure of the membranes. Representative FESEM images of the cross-sections of TON0 and TON1 membranes are displayed in Figure 8. The samples for the FESEM were prepared by freezing pieces of the membranes in liquid nitrogen and fracturing the frozen membrane to observe the cross-sections of the films. The images of the fractured surfaces of both membranes displayed sponge-like porous structures with finger-like walls of PVDF lamellas with uneven pore size distribution. However, it is apparent from the FESEM images that the structure of the pores in TON1 became more open than TON0 due to the presence of citric acid in the dope solution. Consequently, the porosity of the latter is expected to be comparatively higher. This fact is substantiated in the porosity study discussed in the next section.

#### 3.3.5. Porosity Analysis of PVDF Membranes of TONx Series

**Figure 9:**
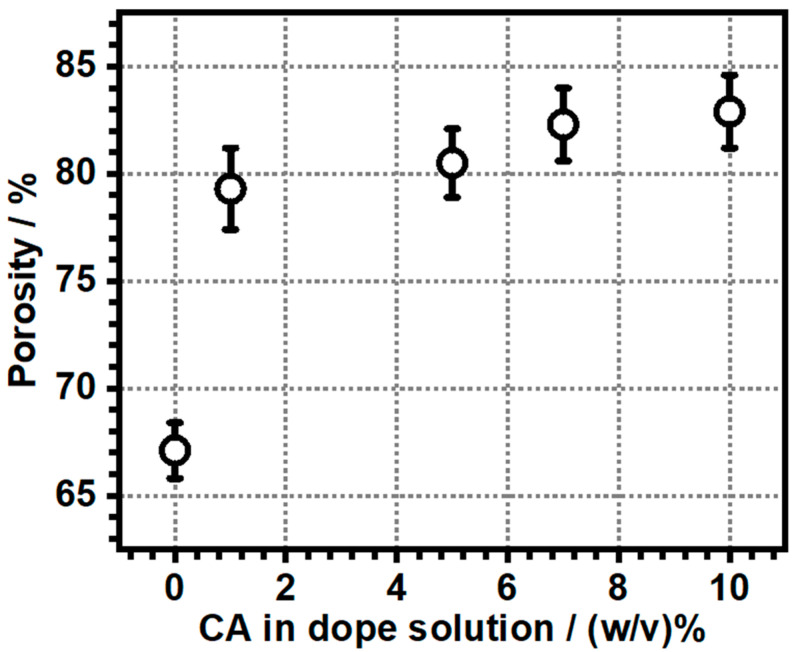
The porosity of the TONx series membranes against the concentration of citric acid (CA) in the dope solution.

We determined the porosity of the membranes gravimetrically by soaking them in hexanol-1. The porosity values are presented in Table 3, and the porosity as a function of citric acid concentration in the dope solution is plotted in Figure 9. The data clearly shows that the porosity abruptly increased by 18% with the addition of 1% CA in the dope solution, after which the porosity linearly increases upon the gradual addition of CA in the dope solution. At the 10% CA addition, the increase in porosity became about 24%.

### 3.4. Morphology and Thermal Stability of PVDF/TiO_2_ (Mx Series) and PVDF/TiO_2_/CA (TAx Series) Membranes

The PVDF-TiO_2_ nanocomposite membranes of the Mx series were prepared following the same procedure as the TONx series membranes by adding a calculated weight of TiO_2_ nanoparticles in the 10% PVDF dope solution in DMAc to make dispersions of 0.0% to 0.4% (*w*/*v*) relative to DMAc in the dope solutions and 0.0% to 4.0% (*w*/*w*) TiO_2_ nanoparticles relative to the PVDF content in the membranes (no citric acid added). Membranes containing more than 4% nano-TiO_2_ could not be prepared because dope solutions with more than 4% nano-TiO_2_ were too viscous and quickly gelled, making them impossible to stir and manipulate. The TAx series membranes were prepared similarly to the Mx series; additionally, the dope solution contained a constant concentration of 5% (*w*/*v*) CA. The composition of the dope solution, the final content of nano-TiO_2_, and the designation of the resulting nanocomposite membrane films are given in Table 2. We then characterized these composite membranes by FESEM, XRD, FTIR, TGA, DSC, and porosity measurements.

#### 3.4.1. X-Ray Diffraction Study of PVDF-TiO_2_ Nanocomposite Membranes of Mx and TAx Series

**Figure 10:**
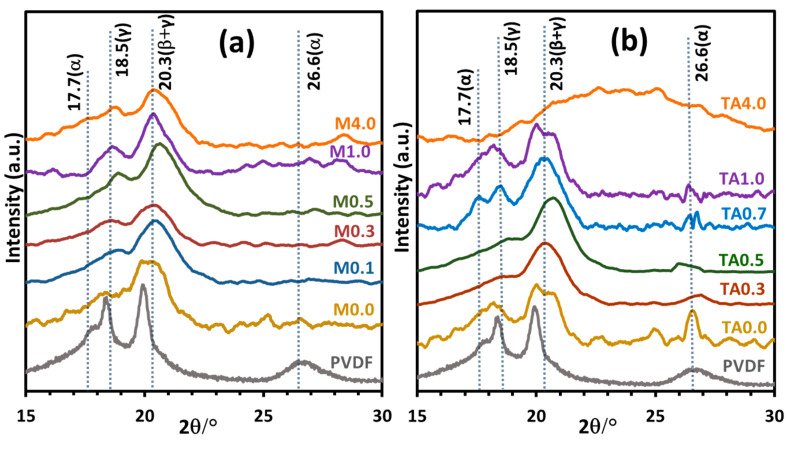
X-ray diffraction patterns of PVDF-TiO_2_ nanocomposite membranes of (**a**) Mx series (no CA in dope solution) and (**b**) TAx series (dope solution contained 5% (*w*/*v*) CA). Pattern marked PVDF represents commercial PVDF powder.

We have displayed the XRD patterns of the PVDF-TiO_2_ nanocomposite membranes of the Mx and the TAx series in Figure 10. In all the membranes, the sharp diffraction features of the α-phase of PVDF powder were absent, but relatively broad peaks featuring the β- and γ-phases evolved. In the M0.0 membrane, diffraction peaks corresponding to the electroactive β- and γ-phases were much more intense than the α-phase diffraction peaks. The XRD patterns of the TAx series membranes displayed a similar nature and trend to those of the corresponding members of the Mx series. We obtained a quantitative estimate of the phase composition from the FTIR data of the membranes, which we have presented in the next section.

#### 3.4.2. FTIR Spectroscopic Study of PVDF-TiO_2_ Nanocomposite Membranes of Mx and TAx Series

**Figure 11:**
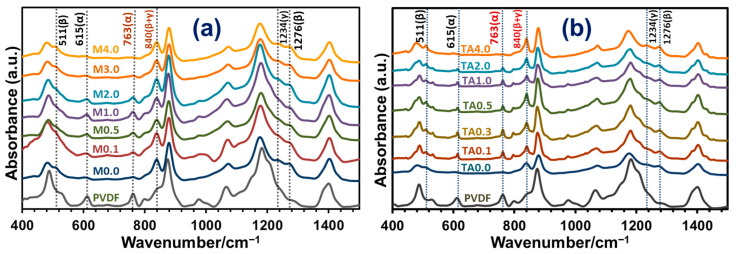
Representative FTIR spectra of PVDF-TiO_2_ nanocomposite membranes of (**a**) Mx series and (**b**) TAx series. Spectra marked PVDF represent commercial PVDF powder.

The FTIR spectra of the neat PVDF powder and the PVDF-TiO_2_ nanocomposite membranes of the Mx and TAx series are displayed in Figure 11a,b, respectively. The intense peak of the neat PVDF powder at 615 cm^−1^ and 763 cm^−1^ corresponding to the α-polymorph decreased significantly in the membranes. In contrast, the absorption peaks at 511 cm^−1^ and 1276 cm^−1^ corresponding to the chain conformation of the β-polymorph and that at 840 cm^−1^ corresponding to the combined β- and γ-polymorphs intensified, which depended on the percentage of nanoparticles in the membrane structure in both series.

**Figure 12:**
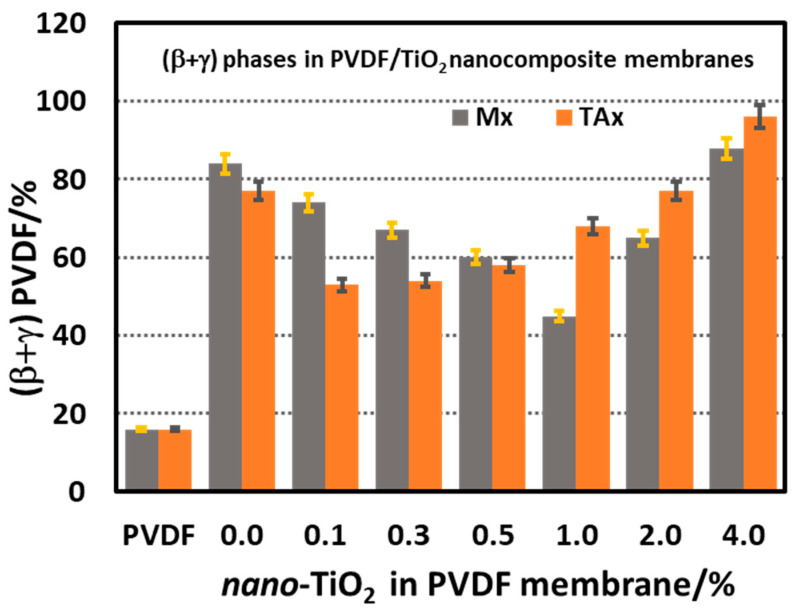
Percentage (±3%) of electroactive (β + γ) conformers of PVDF in Mx and TAx series membranes obtained from FTIR spectra by Gregorio method. FTIR spectra for commercial PVDF powder are also shown as reference.

The percentage of combined electroactive β- and γ-phases of PVDF in the neat PVDF powder and the membranes calculated by the Gregorio method are presented in Figure 12. It was observed that the total percentage of the β- and γ-polymorph conformation of the neat PVDF membrane M0.0 is more than five times higher than that in the neat PVDF powder (16:84), which means that the condition of membrane preparation favors the formation of trans conformation of the chains in the polymer membrane. For the Mx series, the incorporation of 0.0 to 4.0% TiO_2_ nanoparticles in the composite makes the situation quite dramatic: at the lower concentration range of ≤1% nano-TiO_2_, the proportion of the total electroactive component (combined β- and γ-polymorphs) decreased gradually with increasing nano-TiO_2_ content and reached a minimum of about 45%. With a further increase in the concentration of nano-TiO_2_ (>1%) in the membrane, the total electroactive component gradually increased, reaching about 88% compared to the electro-inactive α-phase content.

After adding 5% citric acid in the dope solution (in TAx series membranes), the electroactive (β and γ)-phase content in the TA0.0 (no TiO_2_ nanoparticles) membrane became 77%, which is slightly less than 5 times the neat PVDF powder (77:16). Incorporating 0.1% nano-TiO_2_ at the constant concentration of CA in the dope solution resulted in a drop of the electroactive phase content to 53%; however, further addition of nano-TiO_2_ followed a regular increasing trend and reached up to 96% (β + γ)-phase at 4% nano-TiO_2_ content. When compared to the Mx series membranes, the electroactive phase content in the TAx series membranes remained lower for nano-TiO_2_ containing <1%, beyond which it remained higher for the same level of nano-TiO_2_ content and reached as high as 96% for 4% nano-TiO_2_.

The potential reason for the formation of high electroactive (β + γ)-phase PVDF in the membranes lies in the structure of the dope solution, which we did not study in the present work, nor could we find one in the literature. Recently, PVDF gels containing α-, β-, and γ-polymorphs were studied in detail [83]. For the preparation of the γ-PVDF gel, the authors utilized the NIPS method using a 10 wt% PVDF dope solution in a DMAc-water (4:1 *v*/*v*) mixed solvent, a system much in common to ours, but they did not discuss the mechanism for the formation of the γ-polymorph PVDF. It is known that thermoreversible gelation of semicrystalline polymers in solvents occurs by forming polymer networks whose junction points are crystallites [84]. In work on semidilute solutions of poly(9,9-dioctylfluorene-2,7-diyl) (PF8) in toluene, a good solvent for the polymer, the authors have shown that the solution has a transient network structure formed by the overlap of polymer chains within which aggregate domains of ordered segments are formed (somewhat comparable to the junction points of the polymer gel network, though transient). The chain segments align parallelly within these domains along their long axis [85]. In the present work, the dope solution has a 10 wt% PVDF concentration in DMAc, for which the Flory–Huggins interaction parameter is about −0.6 [84], indicating a strongly interacting system. So, it is possible that in the dope solution, such aggregated domains of aligned segments have formed where chain segments of β (TTTT) and γ (T_3_GT_3_G′) conformation effectively pack. During the NIPS process, these aligned segments (crystallites) in the aggregate domains acted as nucleation sites, resulting in the crystallization of the β- and γ-polymorphs in the membranes with a high proportion (84%). The CA present in the dope solutions disrupted the formation of segmental alignment by interacting with the –CH_2_–CF_2_– dipoles along the polymer chain through its C=O dipole [86], resulting in a reduction (84% to 66%) of the electroactive phase content in membranes prepared from the highest CA content dope solution of the TONx series. It was demonstrated, albeit without a detailed explanation, that TiO_2_ nanoparticles in DMAc solution can nucleate the β-polymorph PVDF-HFP copolymer within the copolymer-TiO_2_ nanocomposites [87]. This observation may suggest that TiO_2_ nanoparticles could play a similar role in our system. However, the exact mechanism by which these additives interact and contribute to the formation and modulation of the β- and γ-polymorph PVDF in the membranes in our specific system is still not fully understood.

#### 3.4.3. Thermal Analysis of PVDF-TiO_2_ Nanocomposite Membranes of Mx and TAx Series

**Figure 13:**
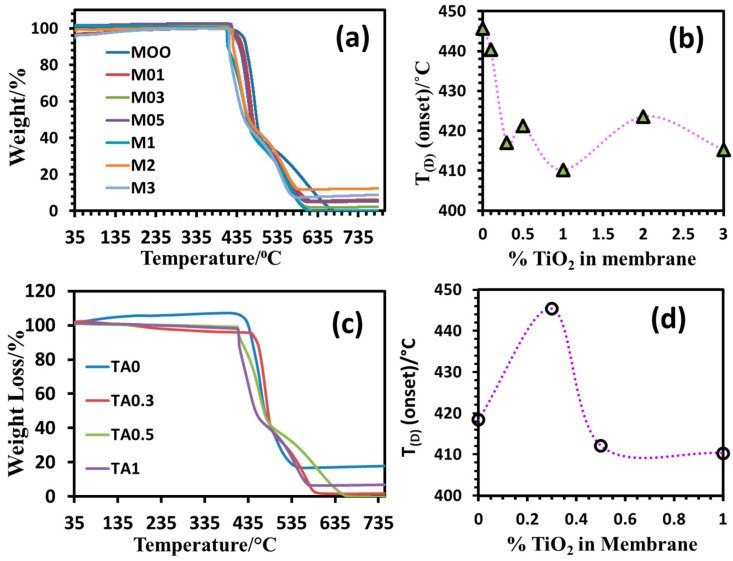
TGA thermograms of the PVDF/TiO_2_ nanocomposite membranes of the (**a**) Mx series and (**c**) TAx series. The samples were heated on the TGA balance from 30 °C to 800 °C under a nitrogen atmosphere at a scan rate of 10 °C/min. A plot of the degradation onset temperature (±0.1 °C) as a function of TiO_2_% in the membrane of the (**b**) Mx series and (**d**) TAx series.

Figure 13a shows that the degradation onset temperature of the neat PVDF membrane was 445 °C, indicating high thermal stability of the PVDF membranes. The weight loss of all the membranes is due to PVDF degradation, for which HF is the primary degradation product. Incorporating TiO_2_ nanoparticles lowered the thermal stability of the membranes of the Mx series compared to the neat PVDF membrane. The results indicate that the –OH groups on the nano-TiO_2_ particle surface might interact with the CH_2_ end of the –CH_2_–CF_2_– dipole of the matrix polymer or that the nanoparticles interrupt the inter-chain attraction, resulting in less compact polymer crystals. Thus, increasing the concentration of TiO_2_ in the membrane structure makes the composite membranes thermally less stable, which, however, almost levels off after adding 0.3% nano-TiO_2_. The membranes of the TAx series displayed degradation characteristics similar to those of the Mx series, except for the 0.3% TiO_2_-containing membrane, for which an abnormal increase in the degradation onset temperature was observed, the reason for which is still unknown. Nevertheless, all the nanocomposite membranes were thermally stable up to 410 °C, indicating their suitability for high temperature applications up to 400 °C.

**Figure 14:**
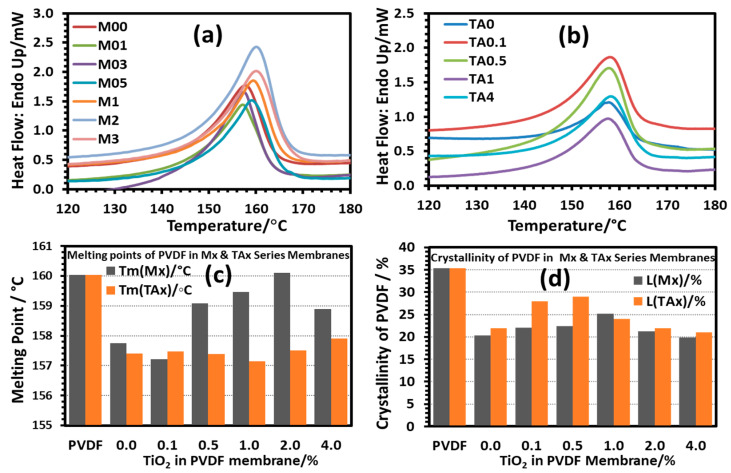
The DSC heating thermograms of as-prepared membranes: (**a**) the Mx series and (**b**) the TAx series. The samples were heated in the DSC furnace from 50 °C to 190 °C under a nitrogen atmosphere at a scan rate of 20 °C/min. (**c**) The melting points (±0.1 °C) and (**d**) the degree of crystallinity (L ± 0.1%) of PVDF in the membranes were determined from the DSC thermograms. PVDF represents the neat commercial PVDF powder used for preparing the membranes.

The melting points and crystallinity of PVDF in the composite membranes were determined by DSC, and the data are presented in Figure 14. For the membranes of both series, the crystallinity data obtained from DSC agreed reasonably well with those obtained from XRD. However, the degree of crystallinity of PVDF in all the prepared membranes decreased significantly compared to that of the neat PVDF powder. It was also observed that the presence of the nanoparticles in the dope solution did not affect the degree of crystallinity of the membrane material that much.

For the Mx series membranes, the melting peak temperature of PVDF decreased significantly by 2.5 °C at a low concentration of the nanoparticles (up to 0.3%), after which the melting point increased and attained the value of the PVDF powder at 2.0% TiO_2_ nanoparticles. Incorporating nano-TiO_2_ >2.0% in the membranes caused a decrease in the melting peak temperature of PVDF. At low concentrations, the TiO_2_ nanoparticles might have been located inside the interlamellar spaces, causing the formation of relatively thinner lamella and making them melt earlier. On the other hand, at higher nano-TiO_2_ concentrations, the nanoparticles might have acted as nucleation sites, causing the quick consumption of nearby PVDF chains, making a low concentration of the polymer chains locally, and forming smaller crystallites, making them melt early.

For the TAx series membranes, the CA present in the dope solution interacted with the –CH_2_–CF_2_– dipole of the polymer chains, hindering the formation of compact lamella during the phase inversion process and causing the formation of lower melting PVDF crystallites. The interaction between the polymer chains and CA might have been so great that the presence of the TiO_2_ nanoparticles could not influence the crystallization process much. Thus, the melting peak temperatures of all the series members remained at the same low level as the concentration of CA was the same in all the dope solutions.

#### 3.4.4. FESEM Study of PVDF-TiO_2_ Nanocomposite Membranes of Mx and TAx Series

**Figure 15:**
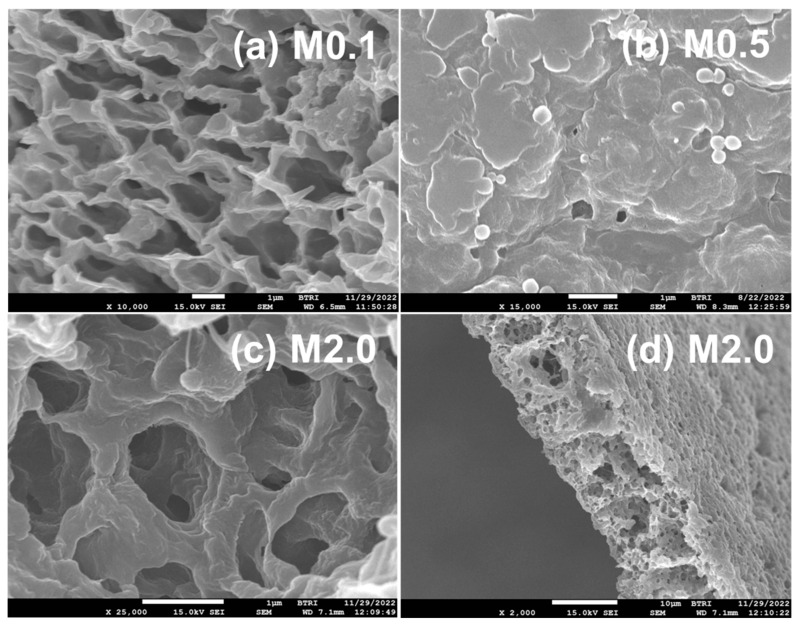
Representative FESEM images of PVDF-TiO_2_ nanocomposite membranes of Mx series: (**a**) cross-section of M0.1 containing 0.1% TiO_2_ nanoparticles, average mesh size is 1.17 ± 0.05 μm (n = 35); (**b**) image of flat surface of M0.5 membrane film, average particle size of nano-TiO_2_ is 268 ± 9 nm (n = 47); (**c**) cross-section of M2.0 containing 2% TiO_2_ nanoparticles, average mesh size is 0.725 ± 0.068 µm (n = 21); (**d**) cross-section of M2.0 membrane, average thickness is 13.23 ± 0.27 µm. Scale bar = 1 μm for (**a**–**c**); and 10 µm for (**d**).

FESEM images of the Mx series nanocomposite membranes shown in Figure 15 demonstrate the specimen’s porous nature, as is typical of membranes. The TiO_2_ nanoparticles incorporated in the membrane matrix are also visible in the images. The size (diameter) of the sphere-like TiO_2_ nanoparticle aggregates measures an average of 268 ± 9 nm from analysis of the FESEM images, whereas the thickness of the membranes averaged at 13.2 ± 0.3 µm. The size of TiO_2_ nanoparticles attached inside the composite membranes was about 1.7 times that obtained from the SEM images of the neat nanoparticles (Figure 1). Careful examination of the spherical entities attached to the surface of the film in the image of Figure 15b would reveal that they have a core–shell type structure, probably formed by winding PVDF chains over the nanoparticles during prolonged stirring of the dope solution, making the particles appear spherical with a much larger size.

When comparing the FESEM image of the neat PVDF membrane shown in Figure 8a (TON0/M0.0) with the TiO_2_ nanoparticle-incorporated membranes of the Mx series in Figure 15, we observed that nanocomposite membranes had compact, rigid-walled honeycomb-like structures with irregularly arranged micrometer-sized chambers and channels. The walls of the chambers/channels appeared to be solid fibrils. The pore structure of the nanocomposite membranes appeared more compact and were expected to have a lower porosity than the neat PVDF membrane (Figure 8a). The latter fact is of outstanding technical significance, which we will substantiate from the porosity measurement of the membranes discussed in the next section.

**Figure 16:**
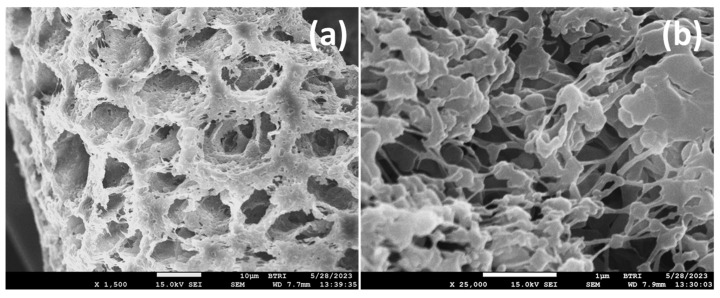
FESEM images of fractured surface of TA4.0 membrane: (**a**) ×1.5 k, average channel size is 10.88 ± 0.37 µm (n = 43), scale bar = 10 µm; (**b**) ×25 k, average mesh size is 0.406 ± 0.024 µm (n = 29), scale bar = 1 µm.

Figure 16 shows representative FESEM images of the fractured surface of the PVDF-TiO_2_ nanocomposite membrane TA4.0 that had 5% citric acid (CA) in the dope solution during preparation. The combined effect of altering the dope solution by CA and incorporating TiO_2_ in the membranes had dramatical consequences on the membrane structure: the channels and chambers became well organized, and the walls of the channels transformed from solid fibrils to a cross-woven nanofiber-like entity, which at higher magnification revealed to have been woven by nanometer-sized spherical polymer particles connected by nanowire-like polymer fibers (Figure 16b). The channel size averaged 10.9 ± 0.4 µm, whereas the mesh size of the channel walls averaged 0.41 ± 0.024 µm.

#### 3.4.5. Porosity Analysis of PVDF Membranes of Mx and TAx Series

**Figure 17:**
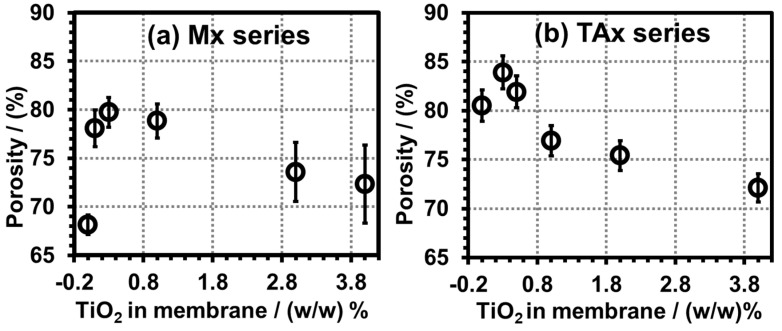
Porosity of PVDF-TiO_2_ nanocomposite membranes as function of nano-TiO_2_ concentration: (**a**) Mx series, and (**b**) TAx series.

Figure 17 displays the porosity versus TiO_2_ particle concentration in the Mx and the TAx series composite membranes. The M0.0 membrane had (68.2 ± 1.0)% porosity, initially increasing to a maximum of (79.7 ± 1.5)% by adding nano-TiO_2_ particles up to 0.3%. Further incorporation of TiO_2_ nanoparticles gradually decreased the porosity of the Mx series and reached 72% at 4% nano-TiO_2_ content. In the case of the TAx series membranes, the dope solutions contained 5.0% citric acid, which raised the porosity of the TA0.0 (no nanoparticle added) membrane to (80.5 ± 1.6)%; the addition of the nanoparticles in the membranes increased the porosity initially up to (83.9 ± 1.7)%, after which the porosity gradually decreased and attained the same level as that of the Mx series membranes, masking the effect of the CA addition in the dope solutions. This observation supports the compactness of the membrane pores and channels at higher nano-TiO_2_ concentrations in the FESEM images of the membranes discussed in the FESEM study.

## 4. Conclusions

This study successfully demonstrates the optimization of the electroactive phase content in PVDF membranes by incorporating citric acid (CA) and nano-TiO_2_ into the dope solution during membrane fabrication via the nonsolvent-induced phase separation (NIPS) method. The NIPS procedure for membrane formation adopted in this study produced highly porous membranes from neat PVDF with high (ca. 84%) β-phase content. Compared to a neat PVDF membrane, the addition of CA and TiO_2_ nanoparticles results in several notable structural morphological changes.

- Structurally, citric acid significantly enhances pore opening, while nano-TiO_2_ transforms sponge-like porous structures into compact, honeycomb-like architectures. The porosity of the membranes increases as a function of additives, reaching 84% with an optimal CA concentration of 5% and low nano-TiO_2_ levels.
- Morphologically, the analysis reveals a significant enhancement in electroactive (β + γ)-phase content due to the presence of additives. In the TAx series membranes, the relative amount of this phase reaches 96%, with the β-phase being the dominant crystalline structure. This enhancement can be attributed to the nucleation of the electroactive PVDF phases from domains of aligned segments formed in the dope solution and a yet unknown interplay of CA and nano-TiO_2_ with the nucleating PVDF domains in the dope solution.

The findings underscore the enormous potential for applying these membranes in energy harvesting, biomedical devices, intelligent scaffolds, and advanced sensors. However, the precise mechanism of additive effects in our system remains unclear. Future research should delve deeper into the nucleation mechanisms in PVDF dope solutions to enhance our understanding of the formation of electroactive phases in the membranes. Additionally, it is essential to investigate the long-term performance and scalability of these optimized PVDF membranes in real-world applications.

## Figures and Tables

**Table 1:** Effect of additives and post-treatments on β-phase crystallinity of PVDF membranes.

Additive	Solvent	Method	% of β-Phase	Reference
CNFs	DMF and Acetone	Solution Casting Followed by Stretching	>96	[51]
PMMA	Dimethicone	Solution Casting Followed by Stretching	93	[17]
dabcoHReO_4_	DMF and Acetone	Solution Casting	95	[52]
MWCNTs	DMF and Acetone	Solution Casting	93.8	[53]
(bmim)[PF_6_]	DMF	Solution Casting and Annealing	92.6	[54]
PZT	-	Solution Casting	57	[55]
ZnO	DMF	Solution Casting	76.1	[56]
TiO_2_/CA	DMAc	NIPS (no post-treatment)	96	This work

**Table 2:** The composition of 10% (*w*/*v*) PVDF dope solutions in DMAc and the designations of the corresponding membranes produced. The number (x) following M and TA in the designations indicates the percentage (*w*/*w*) of TiO_2_ nanoparticles relative to PVDF in the respective membrane films. The number following TON in the TONx series indicates the percentage (*w*/*v*) of citric acid (CA) in the dope solution that was washed out during membrane preparation.

Dope Solution: DS-1		Dope Solution: DS-2			Dope Solution: DS-3	
Designation of Membranes	% Nano-TiO_2_ (*w*/*v*)	Designation of Membranes	% Nano-TiO_2_ (*w*/*v*)	% CA (*w*/*v*)	Designation of Membranes	% CA (*w*/*v*)
M0.0	0.00	TA0.0	0.00	5.0	TON0/M0.0	0.0
M0.1	0.01	TA0.1	0.01	5.0	TON1	1.0
M0.3	0.03	TA0.3	0.03	5.0	TON5/TA0.0	5.0
M0.5	0.05	TA0.5	0.05	5.0	TON7	7.0
M1.0	0.10	TA0.7	0.07	5.0	TON10	10.0
M2.0	0.20	TA1.0	0.10	5.0		
M3.0	0.30	TA2.0	0.20	5.0		
M4.0	0.40	TA4.0	0.40	5.0		

**Table 3:** Degradation onset temperature (T_D_), melting point (MP), degree of crystallinity (λ), and porosity of TONx series PVDF membranes obtained from TGA, DSC, XRD, and porosity measurements.

Sample ID	PVDF ^1^	TON0	TON1	TON5	TON7	TON10
T_D_ (±0.1)/°C	449.1	447.7	441.3	442.6	447.6	442.9
MP (±0.1)/°C	160.0	157.8	158.0	157.4	157.6	157.5
λ (DSC) ^2^/%	35.5	29.4	26.0	21.7	24.0	31.2
λ (XRD) ^2^/%	34	28	20	22	24	26
Porosity ^2^/%	--	67	79	81	82	83

^1^ Commercial PVDF powder used in study. ^2^ Approximate relative error is ±0.1% in λ (DSC), ±2% in λ (XRD), and ±2% in porosity.

## Data Availability

All data are included in the article.
